# The Challenges of the Malaria Elimination Program in the South East of Iran: A Qualitative Study

**Published:** 2019-03-30

**Authors:** Khodamorad Soofi, Narges Khanjani, Fatemeh Kamiabi

**Affiliations:** 1 Department of Epidemiology and Biostatistics, Faculty of Public Health, Kerman University of Medical Sciences, Kerman, Iran; 2 Research Center for Environmental Health Engineering, Faculty of Public Health, Kerman University of Medical Sciences, Kerman, Iran; 3 Department of Environmental Health and Medical Entomology, Faculty of Public Health, Kerman University of Medical Sciences, Kerman, Iran

**Keywords:** Malaria, Elimination, Challenge, Iran

## Abstract

**Background:**

Despite all the efforts made to control and eliminate malaria in Iran, this disease is still considered as a priority health problem in the South East of Iran. We aimed to determine the cultural obstacles which have prevented the elimination of malaria in this region.

**Method:**

This study was carried out through qualitative content analysis. Purposeful sampling was done from people who had malaria or were involved with malaria patients in 2015, in Sarbaz City, Sistan and Baluchestan Province, Eastern Iran. Data were collected through interviews using open questions and continued until data saturation.

**Results:**

The most important barriers in malaria prevention was delay in visiting health centers, delay in diagnosis and treatment due to superstitious beliefs, lack of information about the disease, misdiagnosis and fake doctors. Other obstacles were lack of trust and cooperation with interventions offered by the health system, lack of proper use of the available facilities to prevent malaria and commuting in the high-risk neighbor countries.

**Conclusion:**

Raising awareness in people, officials and health workers about malaria and preventive health interventions as well as health risks associated with fake doctors, following up and re-examination of peripheral blood smear in suspected cases, establishing malaria control stations in border areas and specific measures to refer immigrants and people crossing the border toward malaria diagnosis stations is suggested.

## Introduction

Overall, 212 million cases of malaria and 429 thousand deaths due to malaria have been reported around the world (1). Currently, about 40% of the world’s population who live in low-income countries are at risk of malaria. Although the disease has existed in many parts of the world, today it is mainly found in tropical and subtropical countries and 90% of these cases are in poor African countries (2).

Iran had 799 microscopic confirmed cases that means the total number of cases were 799 and 166 cases were local or autochthonous cases (1). In 2006, 15896 cases of malaria were reported in Iran and the incidence of this disease was 0.2 per thousand people (3) and about 95% of the cases had been reported from three provinces, including Sistan and Baluchestan, Kerman and Hormozgan (4).

Environmental, climatologic, economic, social and cultural conditions and also the province’s shared border with Afghanistan and Pakistan and illegal immigration seem to play an important role in the high number of malaria cases in this province. This province has 1265km shared border with Afghanistan and Pakistan (5). The south and southeast area of Iran is still a troubled region in terms of malaria and it is not comparable with any other part of the country based on social, developmental, economic, cultural, and geographical conditions. These areas are socially similar to underdeveloped and deprived areas (3).

In recent years, there has been a lot of effort to control and eliminate malaria at global level by interrupting the cycle of disease transmission. The World Health Forum identified a 15yr framework for all countries that work on malaria control and elimination in 2015 and called for the elimination of malaria in at least 10 countries by 2020, 20 countries by 2025, and 35 countries by 2030. Less than half of the 91 malaria-prone countries are on track to achieve the 2020 goals of the WHO (1). There is a risk of relapse or re-emergence of malaria in cleared areas and even epidemics in malarious areas of Iran (6). Now, local distribution and transmission of the disease in Iran have been limited to the provinces of Sistan and Baluchistan, Hormozgan and Kerman. The ultimate goal of the malaria elimination program in the horizon of 2025 is to stop local transmission of the disease. Sarbaz City with Annual Parasite Incidence (API) equal to 4.5per ten thousand people in 2015 is a candidate for intensified malaria control operations (7).

About 10% of local (autochthonous) malaria cases in 2015 were from Sarbaz City, which is one of the active foci of malaria in the south-east of Iran. Therefore, there are a series of cultural and social barriers that despite all efforts to control and eliminate malaria, this disease is still an important health problem in the South East of Iran.

Although some qualitative studies have been conducted about malaria control in world countries, no such study has been conducted in Iran, yet. A qualitative study about malaria prevention in Gambia suggested control programs should be conducted at the right time, the target population should be properly educated, sensitization meetings should be held continuously and roles should be defined beforehand (8). In Peru, malaria was believed to be embedded within their culture and people blamed this for lack of attention to prevention. People thought malaria prevention was their government’s responsibility not their own. The other barriers to malaria prevention were people not using bed nets at the right time, not knowing the purpose of space spraying and misconceptions about the cause and prevention of malaria (9). Some mothers in Gabon preferred to consult a Nganga (traditional healer) and not a physician for their children’s malicious fever and thought it was supernatural (10).

This qualitative study was carried out to identify the challenges to control and eliminate malaria in the south-east of Iran.

## Materials and Methods

This research was a qualitative content analysis study conducted in 2015. Participants were purposefully selected from co-operative malaria patients and medical doctors willing to share their experiences and beliefs with researchers. Informed consent was taken from them before the study. Malaria patients were selected from the list of diagnosed malaria cases that were under treatment. Most of these patients were from Sarbaz City. Sampling continued until data saturation was achieved.

Sarbaz City is located in Sistan and Baluchestan Province and because of its high malaria incidence was a suitable place for doing this research. The county has 121km common border with Pakistan and the farthest point is about 250km away from the border. There is no permanent river in this city but suitable larval habitats are present. The surrounding mountains make access to health services difficult for rural people who are 92% of the residents.

Interviews were conducted after participants’ consent. The interviews were recorded and then transcribed. Interviews were done if necessary in local language (Baluchi) and then translated. The average duration of the interviews was 45 to 60min. The questions were semi-structured. The interviewer attempted to understand the experience, views, and beliefs of the interviewees. During the interviews, a better understanding of the situation was achieved and some more questions were added to the interviews. Interviews were conducted until data saturation was achieved.

## Results

Seventeen malaria patients and two medical doctors were enrolled. Six of the patients were drivers who worked across the Iran-Pakistan border (Table 1).

**Table 1.** Some characteristics of the malaria patients participating in this study

**Gender**		**Education**			**Job**				
**Male**	**Female**	**Illiterate**	**Elementary School**	**High School**	**Truck Driver**	**Housewife**	**Farmer**	**Unemployed**	**Labor Worker**
12	5	7	5	5	6	4	2	3	2

The data about the challenges of the malaria elimination program were classified into 4 main groups of concepts.

### Concept 1:

Lack of on-time referral to health services and delays in diagnosis and treatment can be due to several reasons:

#### A. Superstitious beliefs

Superstitious beliefs are one of the most important factors that prevent patients from visiting health care services and therefore by causing delay in diagnosis and treatment lead to persistence and transmission of the disease. One of the malaria patients confessed that when she was very ill and unconscious instead of taking her to a doctor, her relatives burned Espand for her and thought she was stricken by the evil eye. Burning Espand (a desert plant) is believed to prevent the evil eye in superstitions.

“… I was fine, someone cast an evil eye on me, my daughter in law burned Espand for me, but I didn’t get better …”

Some patients especially Falciparum cases (the researcher knew the parasite type from the lab result), after showing severe malaria symptoms, believed that they were haunted by elfs or nasty spirits and needed to see pray drafters, local witches, or religious figures to exorcize the evil spirits. One patient said:

“... I was shivering, I felt bad, my eyes turned white, I felt I was haunted, they took me to the Mullah …”

#### B. Lack of information about the disease

Most patients did not have enough information about malaria, its contagiousness, and prevention to avoid being infected or seek treatment on time. One patient said:

“... First, when a headache and fever started I felt I have a cold, but later when I took the test they said I got malaria! I don’t know how I got malaria?! How should I know that a mosquito bite can cause malaria?! ...”

Another patient said: “... I went to Pakistan and it was very hot there and I was heatstroke … I don’t know how I got malaria when my fever and headache began, I thought it was icter [liver disease] …”

Many patients felt that there is no serious problem and the disease will get better on its own and ignored it, tried home remedies and did not see a doctor which led to delayed diagnosis and treatment. For example, a mother whose child was sick said:

“… First, we gave him a syrup and thought he will get better, after one week still he did not feel better, … later we took him to a doctor …”.

Another patient said: “… When my fever started I thought it is a usual fever and I took fever pills”.

Some of the men said they thought they do not need to visit health centers and health centers are for women, and unless they feel really bad or are under pressure from their families they would not visit the health centers. This ignorance makes the disease worse, causes delayed diagnosis and treatment, leads to persistence of infection in the area and may even cause the patient’s death. For example, one of the people said:

“Here, men do not visit the health centers unless they really felt bad… when I had a fever, I didn’t go to the doctor right away, although my family insisted… on the fourth day, I felt really bad and my family urged me to go to the clinic …”

Also by observing the patients living conditions, the researchers realized that the people living in this area and especially their men are very tough and tolerating. They have grown up in harsh conditions and won’t take a fever serious unless they are well informed about the dangers of malaria.

#### C. Fake doctors

Another reason people do not visit the health care centers is their trust in the fake doctors known as Bengali doctors. Fake doctors are fraudulent people who have no academic degree and attempt to cure patients for money. They are an important threat to public health in this region. A third of the patients that we spoke with had initially visited a fake doctor and then after getting a series of medications and not recovering and feeling worse, eventually visited the health centers.

“The second day because I felt so much worse I went to the Bengali doctor, he gave me two injections, but I didn’t get better, on the fourth day because I got worse my family insisted that I should go to the health center …”

The mother of a sick child said: “First thing, I took my baby to a Bengali doctor and he gave him medicine. He took the medicine for two days but he did not get better …”

Two of the doctors working in Sarbaz City also believed that the presence of Bengali doctors is a threat to public health and complained about the wrong treatments, fake and illicit medications and other harmful treatments.

#### D. False diagnoses

Sometimes, despite the onset of symptoms, due to general and non-specific symptoms of the disease, low number of parasites in blood samples, lab mistakes, and non-native doctors’ that are not aware of the common diseases in the area, early diagnosis of malaria is delayed. A false diagnosis may also keep a patient confident for a long time that his disease is not serious and therefore lead to more delayed diagnosis and more time for disease transmission in the area. A patient said:

“... I went to the clinic because of my weakness and abdominal pain, but the doctor did not even get suspicious that it was malaria and he just gave me a shot and a serum …”

Or another patient said: “I had fever for 4 days, the first day I went to the clinic and they took a blood sample and said you do not have malaria and on the fourth day because I got worse I went to the clinic again and they took another blood sample and diagnosed that it was … malaria and I was taken to the hospital by an ambulance …”

### Concept 2: Lack of trust in interventions done by the health system

Many patients said they did not believe the interventions done by the health care system work. For example, one of the health interventions done in the malaria-prone areas is indoor residual spraying. In order to prevent malaria, people have to believe that this method works and allow proper spraying to be done. But most patients were not fully aware of its effects and considered it useless and futile and were reluctant to cooperate with the healthcare officers. They thought the pesticides are ineffective and the workers who do the job do not have the passion and commitment. Sometimes due to the frustration of repeated pesticide spraying, hot weather, the trouble of unloading home appliances, ruining paint and … people are reluctant to cooperate. Comments of some patients were as follows:

“We spray to kill mosquitoes, but the insecticides don’t work.”

“... Sometimes we don’t feel like spraying, because it is hot and unloading the house is hard …”

“... we did spray insecticides but we did not see any benefit, the insecticides don’t kill anything since there is dust, spraying becomes ineffective ...”

Another patient had a strange thought and said: “… spraying insecticides are useless, the workers don’t spray well … some workers take [steal] the insecticides. They spray water, if they did spray insecticides well, mosquitos would get killed”.

### Concept 3: Improper use of the available facilities to prevent malaria

One of the ways for preventing malaria in the area covered by Sarbaz City health system is the use of insecticide-treated bed nets. However, they are not used and appreciated by the residents for various reasons. People might use this protective tool only when electricity is gone, they have no appropriate cooling system, or when they rest in the yard. For example, one of the patients stated:

“... When electricity is available and air conditioning is on we do not use bed nets; we only use them when power is cut off or when we are sleeping in the yard…”

The small number of people who use the net, do this in order to get rid of annoying insect bites rather than the prevention of malaria. For example, one of the people said:

“... If we don’t use bed nets mosquitos do not let us sleep …”

It was understood from the statements of the participants that even this limited use was not done correctly either. The common time to use mosquito nets in this area is usually after the evening prayers, after having dinner and bedtime, while the *Anopheles* mosquitoes are active from sunset and there is a possibility to get bitten before the time that they actually use their nets. Patients commented that:

“... We have bed nets and we use them when we are going to sleep. We cannot use them from the beginning of night …”

Moreover, in some households, there are not enough bed nets and everyone cannot sleep under the mosquito net.

“… We have bed nets but it is not enough for 4 adults, only little kids sleep under a mosquito net ...”

“… We have bed nets but we don’t use them. It is not enough, for 6 people we only have one net, if the air conditioning is on, we don’t use it …”

But according to the participant’s comments, even in households where there are enough bed nets, they might not be used properly:

“… We are 7 people and we have 4-bed nets, but only 2 or 3 of us use them …”

Participants also mentioned other reasons for not using mosquito nets at the beginning of night and one was the disturbance of livestock.

“... If we use them at the beginning of the night, the goats will tear them apart …”

Moreover, sometimes the mosquito nets they use are defective and there is a risk of mosquito bites.

“… We have bed nets but they have been torn apart …”

These statements can confirm that people have not been well educated about how to properly use and take care of their nets.

In addition, in order for spraying to be efficient, it should be done in accordance with correct principles to have a good performance. But people do not allow their houses to be sprayed for different reasons and they rely on spraying their non-residential premises.

“… If they come for spraying we will let them spray the outdoors, there is no need to spray the indoors…”

### Concept 4: Traveling to high-risk countries

People in this area travel to Pakistan for various reasons, such as treatment of illnesses, seeing their relatives, occupation (such as truck driver), and trade, and become infected by malaria there. Most of the interviewed patients had a history of traveling to Pakistan.

“... I went to Pakistan for 20 days, I am a truck driver and I always travel between Iran and Pakistan”.

“… I am a cross-border driver and I usually travel to Pakistan, I think I was infected in Pakistan because there was no malaria in our village…”

“... I traveled to Pakistan and stayed for a month and a mild fever began there, then I came back to Iran and after 5 nights my fever and chills worsened…”

## Discussion

One way to prevent malaria and reduce the risk of death in patients with this disease is early detection and proper treatment (11–13). One of the expected outcomes of the malaria elimination program for 2025 is diagnosis in less than 24h after the onset of symptoms and starting the treatment within 24h after diagnosis (7).

Our study showed that social and cultural factors are among the factors that have led to malaria elimination failure in the South-East of Iran. Lack of adequate information about the disease, superstitious beliefs and prejudices, not taking the disease and its treatment serious, visiting fake doctors and incorrect diagnoses are among the factors that have led to late diagnosis and treatment and eventually longer opportunities for disease transmission and its persistence in the region.

The role of social factors such as employment status and location of residence in the persistence of malaria in the South East of Iran, has been discussed in previous studies (14). In Ghana, the economic and social situation was significantly related to malaria (15). In Gambia, social factors such as low-quality housing were related to malaria (16). In Minab, Jask and Roudan in Hormozgan Province, Iran; a significant relationship between social factors such as illiteracy and distance from health facilities and the incidence of malaria was observed (17).

Cultural problems such as the low rate of literacy and health education have had a role in the incidence of malaria in Iran (12). This was also mentioned in the case-control study in Chabahar City in which the education level of the control group was higher than the patients with malaria (18). In southern Iran, a direct relationship was found between the level of education and people’s preparedness to participate in the malaria control programs on a voluntary basis (19).

High literacy levels through increased people’s participation in malaria control programs, use of protective equipment, child care, disease knowledge and appropriate job and housing safety can be effective in controlling malaria (18). Implementation of educational intervention programs can increase knowledge, attitude and the practice of preventing malaria in the society (20). The reduced incidence of malaria had taken place for reasons such as an increase in knowledge, attitude and proper performance of people (21). Therefore it seems necessary to plan to increase awareness in the health personnel, public and authorities by local facilities and also take the necessary measures to identify arrest and punish fake doctors who are a threat to public health and take advantage of people nescience to earn money.

The low number of parasite in peripheral blood is one of the most important problems and challenges in the malaria elimination program. A negative blood smear does not mean the patient does not have malaria. Therefore, whenever a person is suspected of having malaria but the blood test is negative, his test should be repeated in the next few days (13) rather than assuring the patient that he does not have malaria.

Lack of people’s trust in the interventions done by the health system is another challenge facing malaria elimination programs. Indoor residual spraying and bed net distribution are among the preventive interventions performed by the Health Network. Although many people are acquainted with spraying and bed nets, in practice, the results of our study and other research show that we are still far away from the appropriate use of these preventive interventions (12, 22, 23).

In this study, some patients commented that the insecticides were not effective. Studies have reported mosquito resistance to insecticides as well (24–26). In order to prevent mosquitoes from developing tolerance, the type of insecticide is changed periodically (23). On the other side, health authorities try to use insecticides that have the least danger for humans. These insecticides might not kill the mosquito immediately, but decrease the mosquito’s lifetime and therefore prevent the transmission of disease. However local people expect not to see any insects at all after spraying and therefore might think the insecticides are not effective.

The results of this study indicate the lack of willingness and cooperation of people for indoor spraying which is the main obstacle against the success of malaria elimination programs (12). In Iranshahr, only 30% of high-risk places were sprayed by insecticides and the reason was residents’ noncooperation despite the fact that most people knew residual spraying was an effective method in reducing malaria transmission. Although more than 95% of Iranians and 90% of Afghan participants knew that spraying, drying swamps and using bed nets prevent malaria but only 24% of participants used mosquito nets and about 90 % of Afghans and 62% of Iranians did not use nets, 77.7% of the participants slept inside and 64.9% of Iranians did not use flyscreens’ for windows (22). This is despite the fact that in Sistan and Baluchestan there are two major malaria vectors including *Anopheles Stephensi* and *An. Culicifacies* that have endophilic (rest indoors) and endophagous (feed indoors) tendencies (27).

Improper use of the existing facilities, despite the high cost and financial burden that it has for the health system, is among the barriers of prevention and eliminating malaria. Several studies have considered using bed nets and spraying, effective to control and prevent malaria (12, 18) and the use of treated bed nets as one of the most promising tools for malaria control in most parts of the world (14). However, misuse of for example window fly screens increases the risk of malaria even up to 5 times (17). The proper use of mosquito nets especially the treated ones requires continuous training to ensure the correct use by the majority of people (18).

Diverting mosquitoes from humans to animals reduces the mosquito-human contact and has a protective role (12) but in case of incomplete and inaccurate spraying, or only spraying outside places and leaving the indoors intact, it will divert the mosquitoes to indoor spaces (13). In order to just satisfy the health authorities, some people allow pesticide spraying in areas such as baths, toilets, animal barns and other places. Spraying these places alone can divert mosquitoes into the residential buildings and people’s sleeping places and therefore trouble malaria control programs.

If the spraying is done properly in place, the abundance of vectors is reduced (28, 29). One of the main features of successful spraying is its comprehensiveness (spraying at-least 70% of all places in the foci), completeness (covering all eligible surfaces) and sufficiency (using the necessary amount of insecticide on surfaces) and repetition at regular intervals (13).

Another challenge for southeastern Iran’s malaria elimination program is its shared borders with high-risk countries. This province has 1265km of shared border with Pakistan and Afghanistan in which legal and illegal movement of people takes place. Among illegal commuters, no supervision or control in regard to their health status is imposed. Many studies have been performed on traveling and migrating between malaria-infected centers and its role in malaria transmission. For example, traveling during the last one month before illness was among the factors that increase one’s risk of getting malaria and people with a history of travel during the last month had a 21 times higher chance of being infected in comparison to others. Chabahar a city near the Pakistan border has been introduced as one of the important foci of malaria transmission (18). Moreover, in the United Arabic Emirates, most malaria cases were Pakistani immigrants (30).

Newly arrived immigrants with less than three months of residence in Iran had a significantly higher incidence of malaria than the ones with more than 3 months of residence (31). The presence of foreigners in this city in 2008, in addition to increasing the number of malaria cases, has led to changes in malaria foci (32) and people traveling from endemic to non- endemic areas had a great role in the outbreak of malaria (33). The important role of migrants in the transmission and outbreak of malaria has been studied by other researchers (34, 35). In Sarbaz County in the former crossing point, a Malaria Passive Post or RDT (Rapid Diagnosis Test) Passive Post was established to discover malaria cases. However, real success is achieved when all local people and officials understand the importance of malaria and cooperate. In addition, since most commuting occurs in other parts of this county (such as Hong, Mortan, Kastag, Kozour, Eshkastag, and Ashar) which is illegal and uncontrolled, planning to put these areas under surveillance seems necessary.

## Conclusion

In order to achieve the goal of eliminating malaria the proper use of existing facilities such as insecticide spraying, mosquito nets, etc., and establishing portable sites for rapid diagnosis of malaria in border areas, is necessary. Emphasis on cross-sectoral and inter-sectoral cooperation, to attract more local health officers who are familiar with the customs and common diseases in the region, and planning long-term educational programs, is also suggested.

Moreover, in order to raise awareness among health professionals, local people and officials; local media, health networking, and the influence of religious leaders should also be incorporated. Holding training sessions in accordance with the culture, to increase public awareness and participation; and penalizing fake doctors who sometimes have the support of local people and local authorities is also necessary.
